# Supplementary material for: The causal relationship between the human gut microbiota and pyogenic arthritis: a Mendelian randomization study
Source: Front Cell Infect Microbiol. 2024 Nov 26;14:1452480. doi: 10.3389/fcimb.2024.1452480 (PMC11629706; doi:10.3389/fcimb.2024.1452480)
Supplement: Supplementary file 1 [file DataSheet1.zip › mendelian test/ebi-a-GCST90027762.csv_forest.pdf]

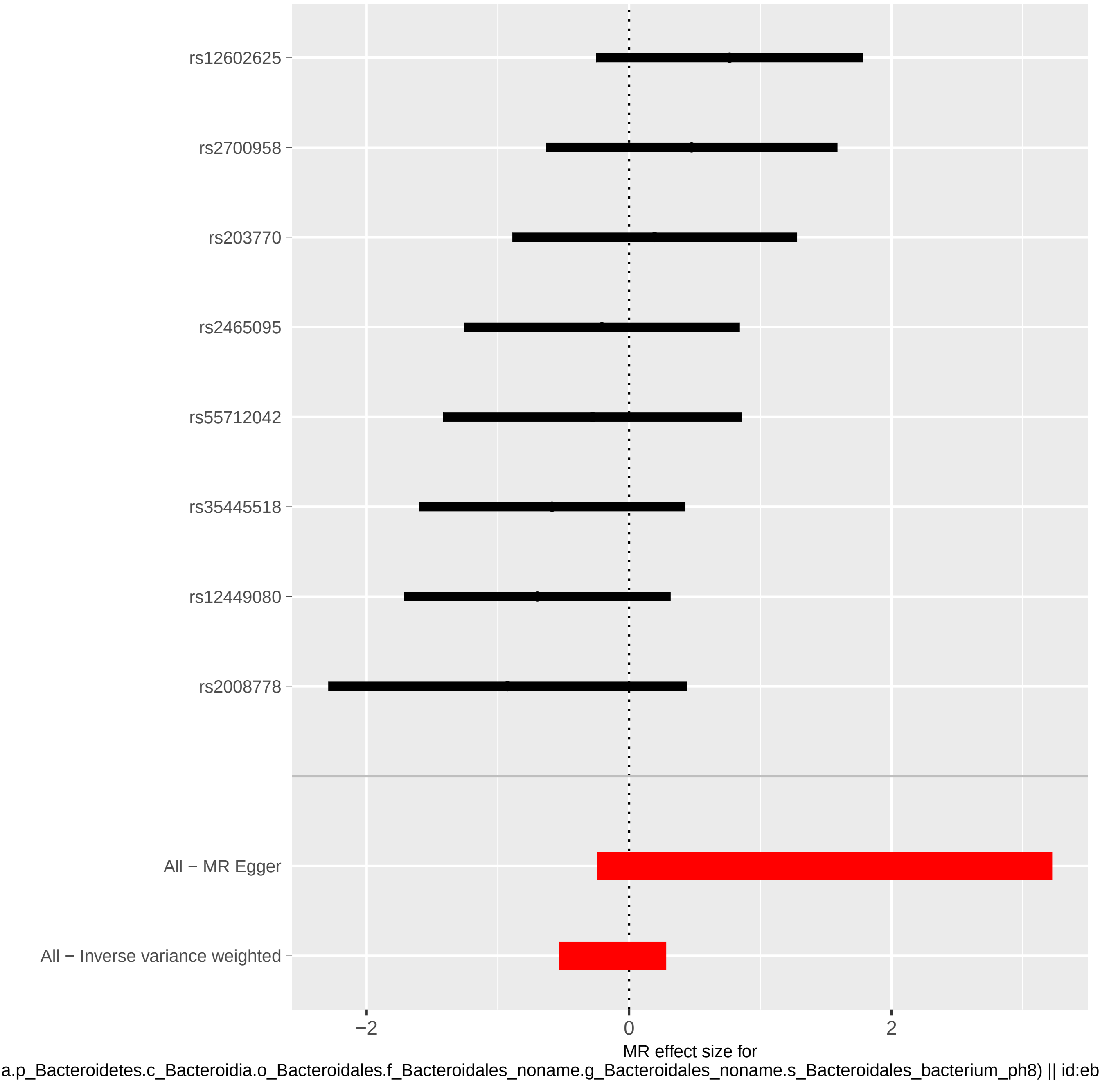
rs12602625
rs2700958
rs203770
rs2465095
rs55712042
rs35445518
rs12449080
rs2008778
All − MR Egger
All − Inverse variance weighted
−2
0
2
MR effect size for
ia.p_Bacteroidetes.c_Bacteroidia.o_Bacteroidales.f_Bacteroidales_noname.g_Bacteroidales_noname.s_Bacteroidales_bacterium_ph8) || id:eb
